# Is that realistic? The development of a realism assessment questionnaire and its application in appraising three simulators for a gynaecology procedure

**DOI:** 10.1186/s41077-018-0080-7

**Published:** 2018-11-08

**Authors:** Erin WILSON, David G. HEWETT, Brian C. JOLLY, Sarah JANSSENS, Michael M. BECKMANN

**Affiliations:** 1 Mater Research, South Brisbane, Queensland 4101 Australia; 2 University of Queensland Faculty of Medicine, Brisbane, Australia; 3 School of Medicine and Public Health, University of Newcastle, Newcastle, Australia; 4 Mater Education, Raymond Terrace, South Brisbane, Queensland 4101 Australia; 5 Mothers Babies and Women’s Health Services, Mater Health, South Brisbane, QLD 4101 Australia

**Keywords:** Simulation, Gynaecology, Medical education, Realism, Simulator design

## Abstract

**Introduction:**

There is no standard approach to determining the realism of a simulator, valuable information when planning simulation training. The aim of this research was to design a generic simulator realism questionnaire and investigate the contributions of different elements of simulator design to a user’s impression of simulator realism and performance.

**Methods:**

A questionnaire was designed with procedure-specific and non-procedure-specific (global) questions, grouped in subscales related to simulator structure and function. Three intrauterine contraceptive device (IUCD) simulators were selected for comparison. Participants were doctors of varying experience, who performed an IUCD insertion on each of the three models and used the questionnaire to rate the realism and importance of each aspect of the simulators. The questionnaire was evaluated by correlation between procedure-specific and global items and the correlation of these items to overall realism scores. Realism scores for each simulator were compared by Kruskal-Wallis and subsequent between-simulator comparison by Dunn’s test.

**Results:**

Global question scores were highly related to procedure-specific scores. Comparison revealed global item subscale scores were significantly different across models on each of the nine subscales (*P* < 0.001). Function items were rated of higher importance than structure items (mean function item importance 5.36 versus mean structure item importance 5.02; *P* = 0.009).

**Conclusions:**

The designed questionnaire was able to discriminate between the models for perceived simulator realism. Findings from this study may assist simulator design and inform future development of a generic questionnaire for assessing user perceptions of simulator realism.

**Electronic supplementary material:**

The online version of this article (10.1186/s41077-018-0080-7) contains supplementary material, which is available to authorized users.

## Introduction

The realism of a simulator is an important consideration in simulation training design [1]. Simulator realism also assists in establishing a fiction contract which along with prebriefing elements such as respect for learners and expectation setting can enhance participant engagement with simulation-based education and create a safe context for simulation-based learning [2]. The level of realism desirable for training depends on the experience of the users, the nature of the simulated procedure to be undertaken, training objectives, cost and the capacity for transfer of learning [3, 4]. Simulator realism has the potential to affect performance following training [5–8] and impact on the acceptability of the simulation to trainees [9]. Knowing what features of a simulator enhance a user’s perception of simulator realism and performance would be valuable when planning simulator design or choosing between existing simulators to achieve a mode of training acceptable to users [4].

When considering acceptable simulator design or selection, it is unclear to what extent a simulator needs to look real (referred to as structural or engineer fidelity) or act real (referred to as functional or psychological fidelity) to appeal to participants. These properties of a simulator are potentially important contributors to perceptions of realism [4]; however, our further understanding of this is hindered by a lack standardisation in assessment. There are a number of procedure-specific simulator realism questionnaires [10–15], and there is a generic simulation experience scale [16]; however, simulator fidelity is only one aspect of a larger template. The development of a simulator realism questionnaire allowing application to multiple procedures may be useful in standardising realism assessment.

The aim of this research was firstly to develop and test a simulator realism questionnaire for its ability to discriminate between simulation models. Global (non-procedure-specific) questions were included in the survey with the aim of evaluating if these questions were similar to procedure-specific questions in relating to participant perceptions of realism (and could potentially be used in a generic simulator realism questionnaire). The final aim was to explore how elements of simulator design contributed to a user’s impression of simulator realism and performance, considering aspects of both structural and functional fidelity.

## Methods

A realism questionnaire was designed, applied and analysed for the technical procedure of IUCD insertion. Insertion of IUCD (intrauterine contraceptive device) was chosen as it is a quick, simple procedure that would reasonably be practiced by doctors of varying experience (to capture a cross-section of clinicians), and multiple simulators are available for the procedure. This study was approved by the institution’s Human Research Ethics Committee to meet the requirements of low- and negligible-risk research.

### Questionnaire design

Questionnaire design was informed by a review of the literature and semi-structured interview of expert IUCD inserters. In this interview, six gynaecology specialists were provided with an example questionnaire and were joined by a study investigator through a detailed discussion of the key steps of IUCD insertion, as well as the features of a simulator considered to be relevant for the procedure. The final questionnaire items were chosen by consensus and pilot tested. Items in the questionnaire were grouped in subscales similar to a previous publication [10]. The subscales included four aspects of simulator anatomical structure (in terms of ‘appearance’, ‘feel’, ‘response to instruments’ and ‘accuracy’ of composition) and five aspects of simulator function (including ‘action’ of the tissue, replication of ‘procedural steps’, ‘vision’, ‘setup’ and ‘perform procedure overall’) (see Additional file 1, a copy of the questionnaire supplied to participants). Additional items in the survey assessed the user’s overall impression of the simulator. These items were the overall realism, value for training and value for assessment. Within each subscale was a global question, designed to be a single non-procedure-specific question to address the same aspects of simulator realism as the procedure-specific questions in the corresponding subscale. Correlation between global and procedure-specific items was intended to assess the content validity of global components of the questionnaire. Additionally, an importance scale was included for realism items in order to capture user perceptions of the importance of each simulator feature in contributing to their assessment of simulator performance.

### Scoring

The questionnaire used 7-point Likert items chosen due to related research [11, 13–15], similar reliability to 5-point scales, to reduce interpolations (in the non-electronic format of the questionnaire) [17] and to capture more sensitive degrees of assessment. [17]

### Simulation models

**Fig. 1:**
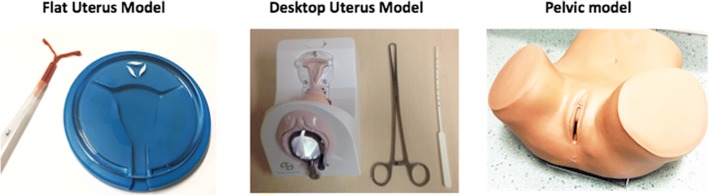
Simulator models

Three simulators were investigated for realism assessment for IUCD insertion (see Fig. 1). Models were chosen that appeared to differ in realism without being obviously superior for training and represented the spectrum of IUCD insertion models available at the institution. The first model, the ‘Flat Uterus Model’, was a clear plastic circular representation of the cervix and uterine cavity allowing visualisation of the IUCD insertion but without further anatomy. The second model, the ‘Desktop Uterus Model’, also contained a clear plastic window into the uterine cavity representation as well as structures representing the vagina and a speculum. The third model chosen, the ‘Pelvic Model’ (ZOE Gynecologic Simulator; Gaumard Scientific®), was an opaque pelvic anatomy simulator (capable of being a simulation model for multiple procedures including IUCD insertion) that included a vulva, vagina, cervix and uterus but not allowing visualisation of the uterine insertion of the device.

### Participants

Participants were doctors working at a large tertiary hospital in Brisbane. Doctors were from a range of experience levels that would reasonably be expected to attempt an IUCD insertion. Doctors were recruited via hospital education sessions. Participants’ role, age and experience are outlined in Table 1.

**Table 1:** Participants

Participant role	Number of participants	Age mean (SD)	Number of previous IUCD insertions		
			≤ 20	21–50	> 50
Medical residents	19	26.9 (4.0)	19	0	0
Obstetrics and gynaecology trainees	17	32.8 (3.5)	5	4	8
Obstetrics and gynaecology specialists	2	42 (2.2)	0	0	2
Total	38	30.4 (5.5)	24	4	10

### Procedure

Doctors were informed of the purpose of the study and given verbal and written instructions for completion. The rationale for the importance scale was explained, and participants were informed how to complete this section in addition to the realism ratings. The doctors performed an IUCD insertion on each of the three simulators and completed the realism questionnaire after each model. Participants performed the IUCD insertions beginning with different models, so there was no set order for the realism assessment. At the end of the three models, participants completed the questions related to the importance of each of the features of the simulator. Questionnaires from participants who were unable to complete all models in the allocated time were kept in the final analysis if all items for a selected model were completed.

### Statistical analysis

#### Realism assessment tool evaluation

Mean scores for all procedure-specific items in each subscale (not including the global question) were determined, to create a ‘mean subscale score’ for each of the nine subscales. The global question scores for each subscale were compared to the mean subscale scores by Pearson’s correlation. Both the global and mean subscale scores were compared to the overall performance score by Pearson’s correlation.

Cronbach’s alpha was used to calculate the interrater reliability in the questionnaire for each model.

#### Assessment of simulator realism

Mean scores for the global items of each subscale, overall realism, value for training and value for assessment items were compared by ANOVA as an omnibus test for significance and if detected, followed with between-model pairwise comparison of means with Sidak correction. ANOVA was used to evaluate the relationship between experience and overall realism scores.

The mean scores for the importance rating in each subscale were used to assess differences in the participant-rated importance of aspects of simulator design.

## Results

The three simulators were assessed by 38 participants who returned 110 realism assessment questionnaires. Four individual simulator blank assessments were excluded.

### Realism assessment tool evaluation

#### Global item correlation

Global question scores were highly related to the mean subscale scores for each corresponding subscale. Pearson’s correlation coefficients were greater than 0.80 (*P* < 0.001) for all comparisons (greater than 0.90 for six of the nine subscales). The global items and the mean subscale scores were both strongly related to the overall realism score (Table 2).

**Table 2:** Correlation of global items to procedure-specific items, overall realism, value for training and assessment

Subscales	Correlation of global item to procedure-specific items	Correlation of global and mean subscale scores to overall realism score	
	Subscale scores *r =*	Global item for each subscale *r =*	Mean subscale score *r =*
1. Appearance	0.87	0.76	0.80
2. Feel	0.90	0.85	0.85
3. Response to instruments	0.91	0.84	0.87
4. Accuracy	0.95	0.88	0.87
5. Action	0.97	0.76	0.76
6. Procedural steps	0.87	0.85	0.80
7. Vision	0.81	0.77	0.83
8. Setup	0.94	0.81	0.86
9. Perform procedure	0.94	0.92	0.86
Overall impression items	Global item correlation to procedure-specific item for each score		
Overall realism	0.95		
Value for training	0.97		
Value for assessment	0.97		

#### Interrater reliability

The interrater reliability for the questionnaire was high, *α* = 0.96 for the Flat Uterus Model, *α* = 0.95 for the Desktop Uterus Model and *α* = 0.93 for the Pelvic Model.

### Realism assessment

Due to the strong correlation between global item scores and procedure-specific items in the subscales as well as overall impression items, global item scores were subsequently chosen to analyse the participant’s perceived realism of the simulators.

#### Subscale scores

Global item realism subscale scores were significantly different across models on each of the nine subscales (*P* < 0.001). Subsequent analysis revealed the Desktop Uterus Model had significantly higher scores than the Flat Uterus Model across all subscales (*P* < 0.001). The Pelvic Model had significantly higher scores than the Flat Uterus Model on all nine subscales (*P* < 0.001) and similar scores to the Desktop Uterus Model in six of the nine subscales (Table 3).

**Table 3:** Realism (global item) scores by model

	Questionnaire scores Mean, standard deviation, median			Comparison by simulator (Sidak correction)		
	1 Flat Uterus Model	2 Desktop Uterus Model	3 Pelvic Model	1v2	1v3	2v3
Realism subscales						
1. Appearance	1.8, 1.4, 1	4.4, 1.3, 5	5.4, 1.2, 5	< 0.001*	< 0.001*	0.005*
2. Feel	1.7, 1.1, 1	4.3, 1.3, 4	4.9, 1.2, 5	< 0.001*	< 0.001*	0.140
3. Response to instruments	1.8, 1.2, 1	4.6, 1.3, 5	4.9, 1.2, 5	< 0.001*	< 0.001*	0.716
4. Accuracy	1.8, 1.1, 1	4.7, 1.1, 5	5.4, 1.2, 6	< 0.001*	< 0.001*	0.017*
5. Action	1.2, 0.5, 1	3.5, 1.7, 3.5	4.0, 1.6, 4	< 0.001*	< 0.001*	0.439
6. Procedural steps	2.5, 1.4, 2	4.9, 1.3, 5	5.6, 1.3, 6	< 0.001*	< 0.001*	0.091
7. Vision	2.9, 1.6, 3	5.1, 1.2, 5	5.5, 1.0, 6	< 0.001*	< 0.001*	0.443
8. Setup	2.1, 1.0, 2	4.2, 1.5, 4	5.0, 1.5, 5	< 0.001*	< 0.001*	0.042*
9. Perform procedure	2.2, 1.3, 2	4.7, 1.2, 5	5.3, 1.3, 6	< 0.001*	< 0.001*	0.209
Overall impression						
Overall realism	1.9, 1.2, 2	4.3, 1.4, 5	5.0, 1.3, 5	< 0.001*	< 0.001*	0.094
Value for training	3.6, 2.0, 4	5.4, 1.3, 6	6.1, 1.0, 6	< 0.001*	< 0.001*	0.129
Value for assessment	2.6, 1.9, 2	4.7, 1.7, 5	5.8, 1.2, 6	< 0.001*	< 0.001*	0.017*

*P < 0.05

#### Overall realism

The overall realism item scores were significantly different across the models (*P* < 0.001), with pairwise comparison revealing the scores for the Desktop Uterus Model and the Pelvic Model were significantly higher than the Flat Uterus Model but a non-significant difference between the Pelvic and Desktop Uterus models (see Table 3).

There was no significant relationship between participant role (*F*(2,105) = 1.39, *P* = 0.25) or number of previous IUCD insertions (*F*(2,105) = 2.48, *P* = 0.09) and scores for overall realism.

#### Simulator performance for training and assessment

There was a significant relationship between simulators and performance for training and assessment item scores (*P* < 0.001). The Pelvic Model and Desktop Uterus Model received significantly higher scores for value for training than the Flat Uterus Model (see Table 3), with no significant difference between the Pelvic and Desktop Uterus Models. For the assessment item, the scores for the Pelvic Model were significantly higher than those in both the Flat and Desktop Uterus Models (see Table 3).

#### Importance scale

**Fig. 2:**
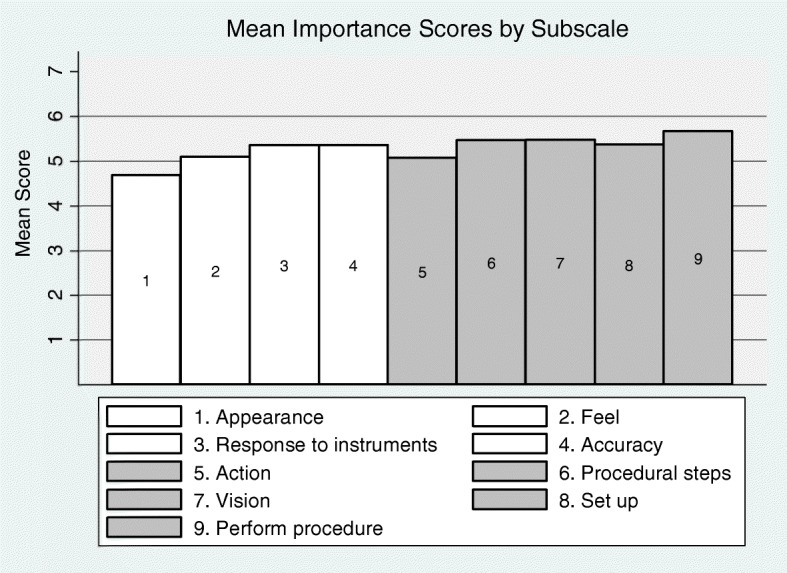
Importance scores for simulator features (1–4 structure items, 5–9 function items)

Importance scale data was returned blank for ten participants. Lower importance scores were found for structure items related to the realism of the appearance of the anatomy and the feel of the anatomy (Fig. 2). The realistic action of the tissue was the lowest rated function item. Higher importance scores were related to function items, such as the ability of the simulator to be realistic in performing the procedure overall, providing realistic vision and procedural steps. The mean importance of subscales 1–4 (structure subscales) was lower than the mean of subscales 5–9 (function subscales; 5.02 versus 5.36, *P* = 0.009).

No significant differences were found between the importance score ratings of participants and different experience levels (by role or number of previous insertions, data not shown). Blank responses precluded intergroup analysis by number of previous insertions.

## Discussion

The designed questionnaire appeared valid in discriminating between simulators and demonstrated high interrater reliability. There was a strong correlation between the global items and procedure-specific items across subscales and in overall realism, value for training and assessment, providing support for the use of a generic questionnaire.

Results from detailed analysis of the global items demonstrated that participants perceived the realism of the simulators to be different across the chosen models. The Pelvic Model was rated highest in realism for the majority of subscales, in overall realism and value for training and assessment. The Flat Uterus Model was considered the least realistic model. These findings provide support for the content validity of the questionnaire and demonstrate the capacity of the questionnaire to discriminate between simulators. The Desktop Uterus Model was considered to be of similar realism to the Pelvic Model in many of the subscales, despite the vast apparent differences in simulator design. Perhaps the similar realism scores were due to the ability to visualise the placement of the IUCD in the Desktop Uterus Model, which may have scored favourably as it provided additional feedback considered valuable for training, possibly also explaining the similar scores for training value. The Flat Uterus Model, which had the lowest scores for overall realism, value for training and assessment, also demonstrated the opening of the device but lacked additional anatomical structures, which may have conferred an overall negative opinion of the simulator that influenced further aspects of its evaluation. The high realism scores for the Pelvic Model may be explained by its human body representative appearance, with literature suggesting participants seem to favour simulations of higher fidelity [18, 19]. This preference may explain the significantly higher scores received for the assessment value of the Pelvic Model compared with the Desktop Uterus Model, which otherwise had similar realism scores. Participants may also have felt the Pelvic Model provided a fairer means of assessment, as no additional visual feedback could be gained.

There was no significant relationship found between experience (level or number of previous procedures) and overall realism scores, demonstrating that participants of differing experience viewed the simulators similarly. There are suggestions that performers of differing experience levels may benefit from different levels of simulator fidelity [1, 20], yet it is apparent that their assessment of simulator realism is similar.

The importance scale scores revealed that items related to the function of the simulator had higher scores than items related to the structure (anatomy) of the simulator. This finding is consistent with arguments that simulator function may be more important than appearance [4, 7]. One of the function items, the ‘action of the simulator in response to the procedure (e.g. tearing, bleeding)’ was rated lower than the other items in this category, possibly as this item was more closely related to simulator structure or that such a function was not relevant to this particular procedure of IUCD insertion. The realism of performing procedural steps and the realism of viewing steps of the procedure in the simulator were rated high in importance. This information may be useful in the design or selection of IUCD insertion simulators. The findings may support a focus on function over appearance in simulator design in a wider range of procedures if similar results were found in future research.

This study has assessed simulator realism through user perceptions of realism and performance of the simulator, which is a limitation of the study as there was no capacity to objectively quantify the realism of any simulator. Global realism questions were of demonstrated value for discriminating between simulators, but it must be considered whether the proceeding procedure-specific items influenced global item scores. The wording of additional items may have caused the participants to consider the features of the simulator more carefully in their assessment of realism [10]. Realism assessment is also only one factor to consider in the overall utility of a simulator. The findings of this study do not allow an assumption of a relationship between this realism assessment and performance outcomes following simulator training. Additionally, the realism questionnaire was only applied to models for the procedure of IUCD insertion, and the features of the simulator deemed important may differ for other procedures. Future evaluation of a generic questionnaire in a variety of simulators would be desirable.

## Conclusion

This study has demonstrated that a realism assessment questionnaire can be used to discriminate between models for user perceptions of simulator realism. Global questions provided in the questionnaire were highly correlated to the procedure-specific items and were similar to the procedure-specific items when correlated to the overall realism scores. Participants considered simulator design components related to the function of the simulator of greater importance than structure components. It is hoped that these findings assist simulator design and future development of a global questionnaire for assessing user perceptions of simulator realism.

## Additional file

Additional file 1: ᅟQuestionnaire. (PNG 338 kb)

## Abbreviations

ANOVA: Analysis of variance
IUCD: Intrauterine contraceptive device
