# Supplementary figures and images for: Is that realistic? The development of a realism assessment questionnaire and its application in appraising three simulators for a gynaecology procedure
Source: Adv Simul (Lond). 2018 Nov 8;3:21. doi: 10.1186/s41077-018-0080-7 (PMC6225559; doi:10.1186/s41077-018-0080-7)

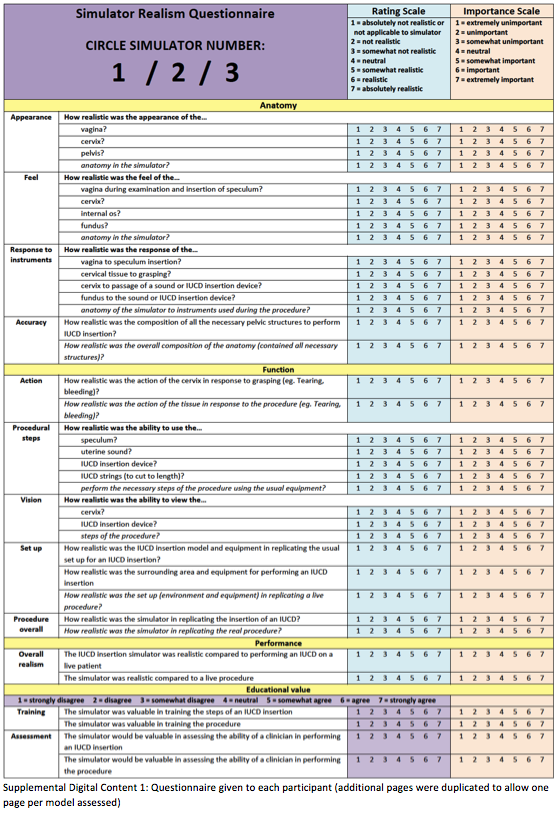

Supplement: Supplementary file 1 — ᅟQuestionnaire. (PNG 338 kb) [file 41077_2018_80_MOESM1_ESM.png]
